# The N-Terminal Region of the Human Autophagy Protein ATG16L1 Contains a Domain That Folds into a Helical Structure Consistent with Formation of a Coiled-Coil

**DOI:** 10.1371/journal.pone.0076237

**Published:** 2013-09-24

**Authors:** Rhiannon Parkhouse, Ima-Obong Ebong, Carol V. Robinson, Tom P. Monie

**Affiliations:** 1 Department of Biochemistry, University of Cambridge, Cambridge, United Kingdom; 2 Department of Chemistry, University of Oxford, Oxford, United Kingdom; University of Edinburgh, United Kingdom

## Abstract

Autophagy is a fundamental cellular process required for organelle degradation and removal of invasive pathogens. Autophagosome formation involves the recruitment of, and interaction between, multiple proteins produced from autophagy-related (ATG) genes. One of the key complexes in autophagosome formation is the ATG12-ATG5-ATG16L1 complex. ATG16L1 functions as a molecular scaffold mediating protein-protein interactions necessary for formation of the autophagosome in response to both classical and pathogen-related autophagy stimuli. The coiled-coil domain of the yeast ortholog, ATG16, exists as a homodimer both in solution and in the crystal form. The yeast and human orthologs show poor sequence identity. Here we have sought to determine the minimal boundaries of the human ATG16L1 coiled-coil domain and ascertain its oligomeric status in solution. Using a range of biochemical and biophysical techniques we show that the secondary structure of the human ATG16L1 coiled-coil has the expected helical composition and that the domain forms a homodimer in solution. We also observe extensive sequence conservation across vertebrates providing strong support for the crucial functional role of the ATG16L1 coiled-coil.

## Introduction

The degradation of cellular material is an important homeostatic function that enables the removal of redundant, broken and potentially harmful material; whilst simultaneously increasing resource availability in the cell. Two key components of cell degradation pathways are the proteosome and the process of macroautophagy (referred hereafter as autophagy). In autophagy, the formation of a double-membrane autophagosome around cellular targets such as damaged organelles, or invasive micro-organisms, facilitates subsequent fusion with lysosomes and the breakdown of the material within the autophagosome. The formation of an autophagasome requires a hierarchical series of interactions between both individual autophagy proteins and preformed protein complexes. ATG16L1 is central to this process, forming part of the ATG12-ATG5-ATG16L1 complex, which is required for the recruitment of LC3 (ATG8 in yeast) to the autophagosome [1]. Removal of ATG16L1 abrogates the ability of cells to form autophagosomes [2].

The N-terminus of ATG16L1, and its yeast ortholog ATG16, is responsible for inclusion of ATG16L1 in the ATG12-ATG5-ATG16L1 complex via interaction with two ubiquitin-like fold domains in ATG5. The molecular basis of this interaction has been determined for both the yeast and human systems and highlights the importance of a helical segment of ATG16L1/ATG16 [3], [4]. Two recent reports both identified FIP200 (focal adhesion kinase family interacting protein of 200 kDa), a member of the ULK1 (UNC 51 like kinase 1) autophagy complex along with ULK1, ATG13 and ATG101, as a direct binding partner of ATG16L1 [5], [6]. The interaction between FIP200 and ATG16L1 allows recruitment of the ATG12-ATG5-ATG16L1 complex to the ULK1 complex at the site of the assembling autophagosome. The critical involvement of ATG16L1 as a key mediator of essential protein interactions required for autophagy is highlighted by the recruitment of ATG16L1 to the site of bacterial invasion by the pattern recognition receptors NOD1 and NOD2 [7]–[9]. This interaction requires the CARD of NOD1 and NOD2 and the WD40 repeats of ATG16L1 [10], [11]. In the case of NOD2 the interaction involves a newly reported 19-amino-acid ATG16L1-binding motif also found in TLR2 (Toll-like receptor 2), T3JAM (TRAF3 interacting protein 3), DEDD2 (death effector domain containing 2) and transmembrane protein 59 (TMEM59) [10]. Currently it is unclear whether all these proteins play an active role in autophagy. However, at least in the case of TMEM59 the interaction with ATG16L1 mediates the degradation of its own endosomal compartments and enables a protective autophagic response to *Staphylococcus aureus* infection [10].

In addition to mediating heterotypic protein interactions ATG16L1 also undergoes homotypic interactions via its coiled-coil domain. The structure of the coiled-coil domain of the yeast ortholog, ATG16, revealed the formation of a parallel dimeric coiled-coil. Coiled-coils are found in almost all areas of cell functionality and are common protein interaction surfaces formed between extended amphipathic helices. Numerous oligomerisation states have been observed for coiled-coils, with dimers, trimers and tetramers the most common [12]. In addition to yeast ATG16 coiled-coils have also been reported for other autophagy proteins including Beclin-1 [13], FIP200 [14] and ATG11 [15]. In this work we have expressed and characterised the coiled-coil domain of human ATG16L1. We show that it folds as a helical protein and exists as a dimer in solution, consistent with the structural information from the yeast ortholog ATG16. A crucial role for the ATG16L1 coiled-coil in complex formation is supported by an extremely high level of sequence conservation between vertebrate species.

## Results and Discussion

### Expression of the ATG16L1 coiled-coil domain

**Figure 1:**
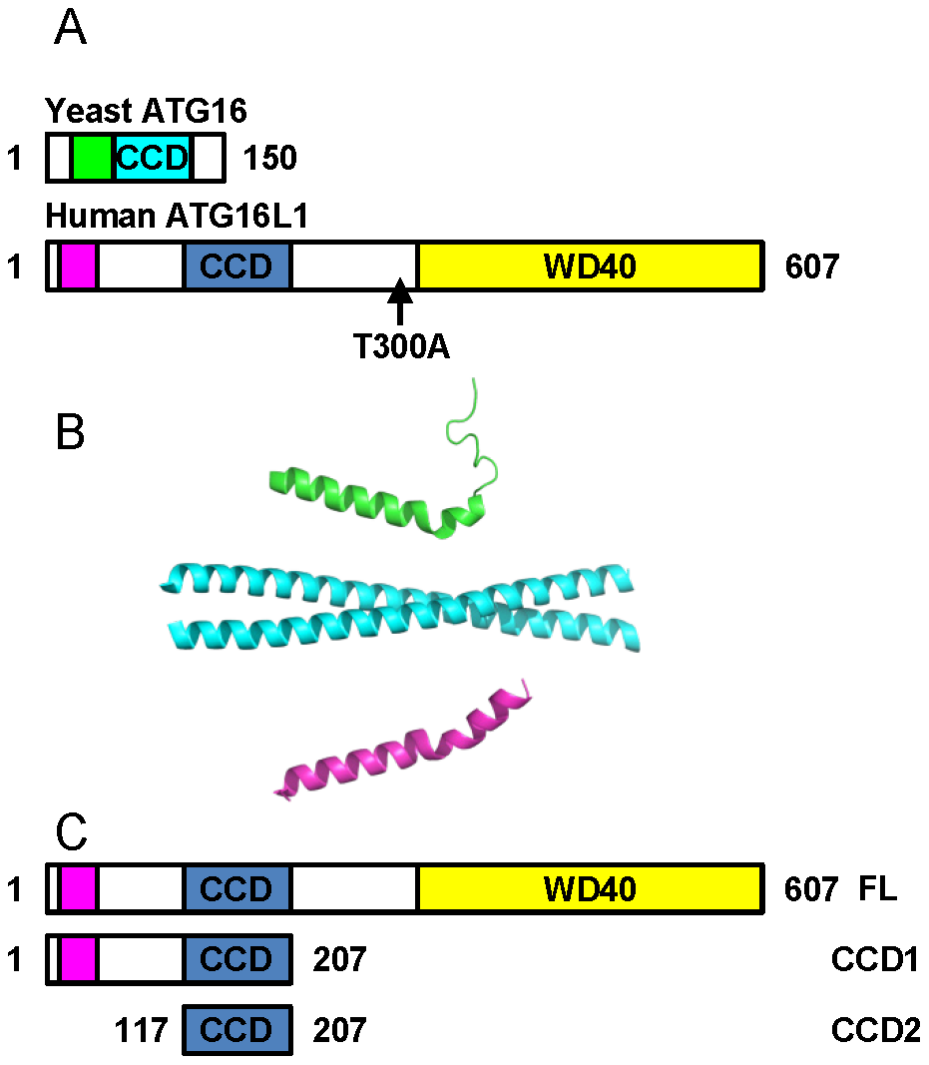
Organisation of ATG16L1. (A) Domain organisation of yeast ATG16 and human ATG16L1. ATG5 binding motifs are coloured green (yeast) and pink (human); the coiled-coil domain (CCD) is cyan (yeast) and blue (human); the human WD40 repeats are yellow. The position of the Crohn's Disease susceptibility polymorphism T300A is marked on human ATG16L1. (B) Structure of the yeast and human ATG5 binding motifs and the yeast ATG16 CCD. Colours as in panel (A). (C) Expression constructs used in this study outlining the boundaries of human ATG16L1. FL – full length ATG16L1, CCD1 – coiled-coil domain construct 1, CCD2 – coiled-coil domain construct 2.

Although functionally similar distinct differences exist in the domain organisation of yeast ATG16 and mammalian ATG16L1 (Figure 1A). Studies of ATG16 from *Saccharomyces cerevisiae* have shown that the protein possesses an ATG5 binding motif at its N-terminus, followed by a coiled-coil domain. Both these domains have previously been successfully crystallised (Figure 1B). The human form, ATG16L1, also contains an N-terminal ATG5 binding motif. However, unlike the yeast protein, this is followed by an extended linker region leading into a coiled-coil, a second linker region, and a series of WD-40 repeats (Figure 1A).

There is limited sequence homology between the coiled-coil regions of human ATG16L1 and yeast ATG16. A search of the NCBI non-redundant protein sequence database with the coiled-coil of *S. cerevisiae* ATG16 failed to return any significant hits when limiting results to proteins from *Homo sapiens*. Despite this limited primary sequence homology Fujioka and colleagues were previously able to align the two proteins on the basis of a pattern of repeating hydrophobic residues in the *a* and *d* positions of the helix (Figure 1 in [16]). We used this alignment as a basis for the design of three initial expression constructs containing the human ATG16L1 coiled-coil domain (Figure 1C). These were: full-length ATG16L1 spanning residues M1-Y607 (FL); residues M1-A207 containing the ATG5 binding motif, the first linker region and the coiled-coil (CCD1); and residues M126-A207 encompassing the minimal coiled-coil domain proposed by the alignment with yeast ATG16 (CCD2) (Figure 1C). All constructs were screened for expression with a variety of N-terminal fusion tags: 6-His alone; GST (glutathione S-transferase); 6His-NusA (N utilisation substance protein A); and 6His-MBP (Maltose binding protein). Each construct also possessed a C-terminal FLAG-6-His epitope tag. Full-length protein was entirely insoluble. However, CCD1 and CCD2 expressed with each tag except the 6-His tag alone (Table 1). Expression levels were comparable between fusion partners so the GST fusion constructs were selected for large scale expression and purification as GST is simple and effective to use and has been previously used to successfully purify yeast ATG16 [17].

**Table 1:** Summary of human ATG16L1 construct expression in *E. coli* Rosetta™ 2 cells.

N-terminal tag	Human ATG16L1 construct		
	FL (M1-Y607)	CCD1 (M1-A207)	CCD2 (M117-A207)
6His	Insoluble	Insoluble	Insoluble
GST	Insoluble	Soluble	Soluble
6His-NusA	Insoluble	Soluble	Soluble
6His-MBP	Insoluble	Soluble	Soluble

Footnote: All constructs possessed a C-terminal FLAG-6His epitope tag. Abbreviations: 6His – 6× Histidine; GST – Glutathione-S-transferase; 6His-NusA – 6× Histidine N utilisation substance protein A; 6His-MBP – 6× Histidine Maltose binding protein.

### Purification of the ATG16L1 coiled-coil domain

**Figure 2:**
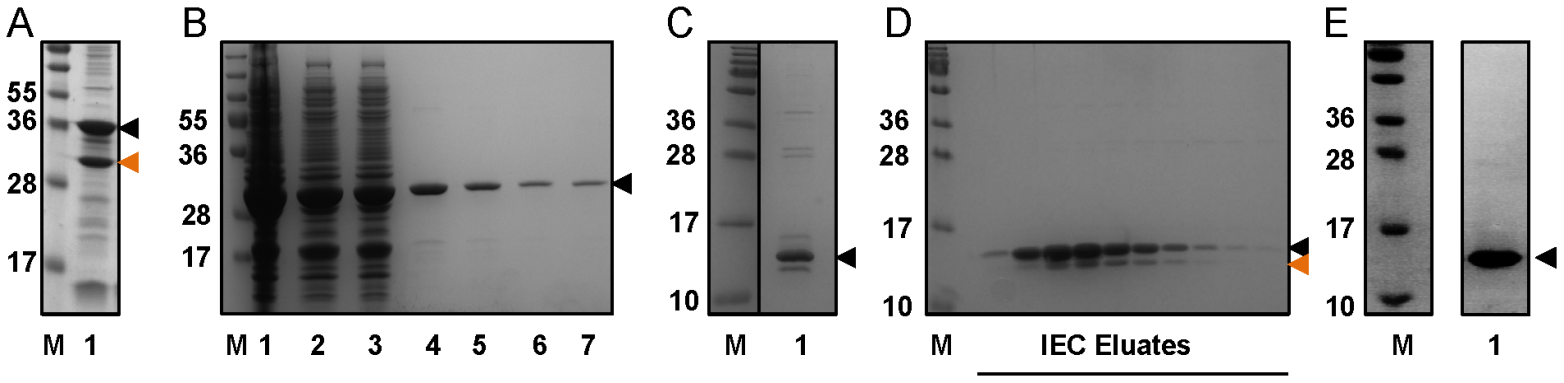
Purification of recombinant human ATG16L1 coiled-coil. (A) GST-CCD1-FLAG-6His expression produces a truncated product (orange arrowhead). (B) Glutathione sepharose purification of GST-CCD2-FLAG-6His. 1 – total cell lysate, 2 – soluble extract, 3 – unbound flow through, 4–7 successive elution fractions. (C) CCD2-FLAG-6His following removal of the GST tag by TEV cleavage (lane 1). (D) Elution fractions of CCD2-FLAG-6His after anion exchange. The position of the truncated protein is marked by an orange arrowhead. (E) CCD3-FLAG-6His (lane 1) is highly pure and shows no evidence of truncation following glutathione sepharose affinity purification, TEV cleavage and hydrophobic interaction chromatography. In all panels the black arrowhead marks the bands representing the expected size of the ATG16L1 construct; M denotes PageRuler™ Plus Prestained protein standards (Thermo Scientific).

CCD1 and CCD2 were both purified by GST pull down, on column TEV cleavage and anion exchange (Figure 2). SDS-PAGE analysis of both recombinant proteins indicated the presence of truncated, or cleaved, protein products (Figure 2A and 2D). Analysis by mass spectroscopy and N-terminal sequencing indicated that the CCD1 truncation had lost the first 55 amino acids and now began at L56 within the linker region. The truncated CCD2 protein had been cleaved between Q125 and M126. Given the observed cleavage immediately before M126, we designed a new construct (CCD3) spanning residues M126-A207. CCD3 was expressed as a GST-CCD3-FLAG-6His fusion and purified to homogeneity using glutathione sepharose, on column TEV cleavage, and HIC (Figure 2E). CCD3 showed no evidence of truncation indicating the formation of a stable protein and was selected as the final construct for further characterisation.

### The ATG16L1 coiled-coil adopts a helical conformation

**Figure 3:**
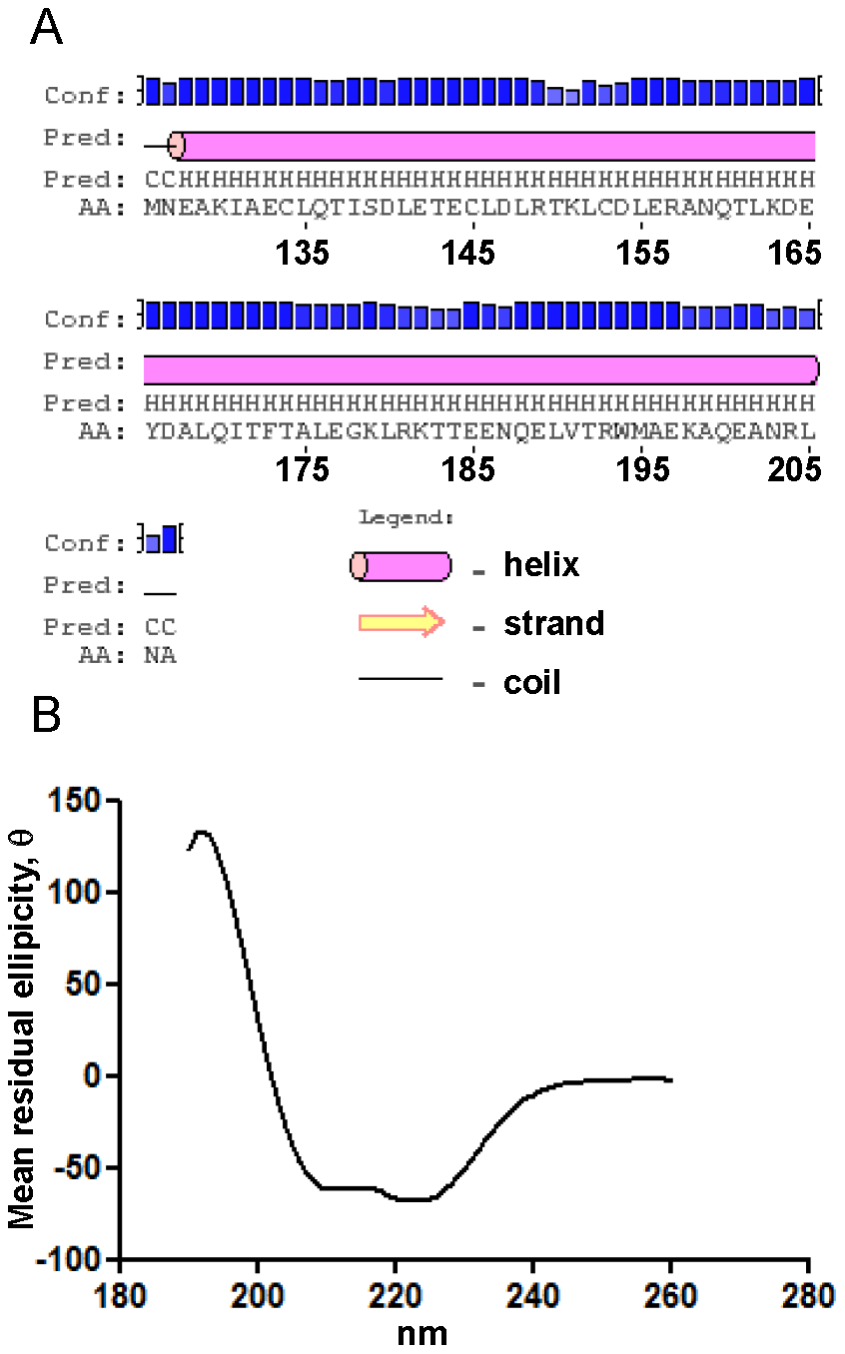
The coiled-coil of human ATG16L1 is alpha helical. (A) PSIPRED prediction of the secondary structure of the minimal coiled-coil domain (residues M126 – A207; CCD3) of human ATG16L1. Sequencing numbering corresponds to the human protein, alpha helices are marked as pink cylinders and the confidence level of the prediction for each residue is provided by the blue bars. (B) Circular dichroism analysis of CCD3. Mean residual ellipicity (θ) plotted against wavelength (nm) indicates a helical protein.

The region of ATG16L1 (M126-A207) encompassed by our minimal CCD3 construct closely correlates with the section aligned to the coiled-coil of ATG16 by Fujioka and colleagues [16]. Consistent with this the secondary structure of CCD3 (M126-A207) was predicted to be entirely alpha helical by the PSIPRED server (Figure 3A). The helical nature of CCD3 was confirmed by circular dichroism (Figure 3B). SELCON3 analysis revealed the protein to be approximately 80% helical, 5% turns and 15% disordered. Together these results provide strong indication that the core, stable, and folded portion of the coiled-coil domain of human ATG16L1 is found between residues M126 and A207.

### Analysis of the multimeric nature of the ATG16L1 coiled-coil

Yeast ATG16 was originally reported to homo-oligomerise in a process dependent on the coiled-coil domain [18]. Analysis of the complex formed between yeast ATG16, ATG5 and ATG12 suggested a molecular weight of approximately 350 kDa, for which a tetrameric assembly was postulated [19]. The murine ortholog, which, like the human protein, contains WD40 repeats, was suggested to exist in an octomeric assembly with murine ATG5-ATG12 conjugates following detection of an approximately 800 kDa complex of ATG16L1-ATG5-ATG12 from murine cells [20]. In the isolated form yeast ATG16 exists as a dimer both in the crystal structure and in solution as determined by analytical ultracentrifugation [16].

**Figure 4:**
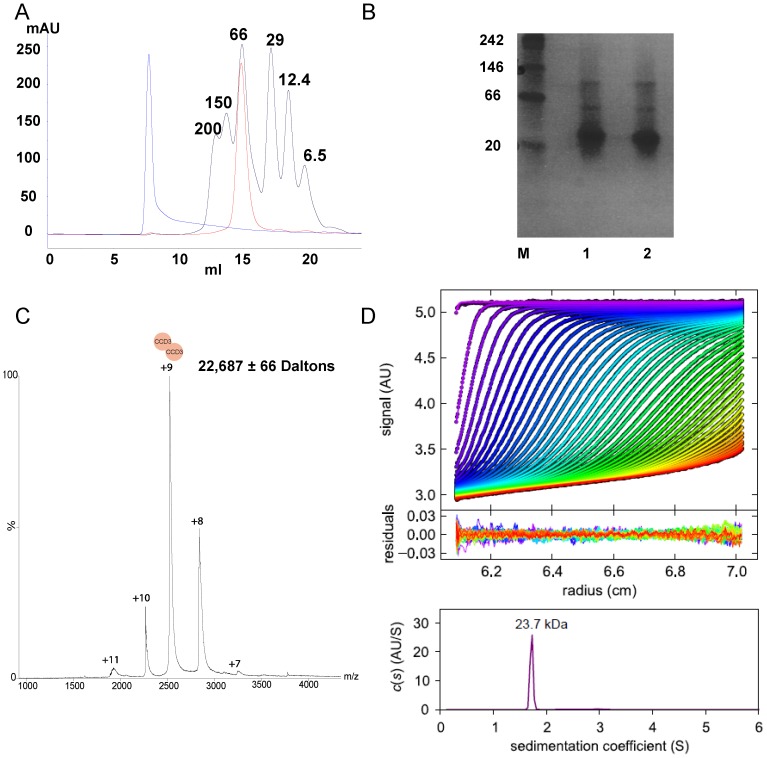
Characterisation of the oligomeric status of the human ATG16L1 coiled-coil domain. (A) Size exclusion chromatography of CCD3. CCD3 – red trace; Blue Dextran – blue trace; Molecular size markers – black trace (mass labelled in kDa). (B) Native-PAGE analysis of CCD3. 1 µg of CCD3 was analysed by Native-PAGE both before (lane 1) and after (lane 2) freeze/thawing at −80°C. M – NativeMark™ protein standards (Life Tehnologies). (C) Nanospray Electrospray Ionisation Mass Spectroscopy (ESI-MS) analysis of CCD3 under conditions that preserve non-covalent interactions. CCD3 dimers are detected in the low m/z range corresponding to charge states of +11 to +7. There is no evidence of any additional species of CCD3. (D) Analytical ultracentrifugation sedimentation velocity data. Interference optical signal distributions of CCD3FH at time intervals of 320 s at a rotor speed of 50,000 rpm and a temperature of 20°C, with systematic noise subtracted. The residuals are from the fit with the hybrid discrete/continuous model. Component sedimentation coefficient distributions showed a dominant population of the dimeric species, with a uniform frictional ratio of Fk,w = 1.73. The final r.m.s.d. was 0.006.

To investigate the oligomeric state of human ATG16L1 CCD3 in solution, we first analysed the recombinant protein using two standard techniques, size exclusion chromatography (SEC) and Native-PAGE. Based on its amino acid sequence the calculated molecular weight of CCD3 is 11.3 kDa. SEC produced a single symmetrical peak (Figure 4A) with an estimated molecular mass of approximately 70 kDa, suggesting that human ATG16L1 is a hexamer in solution. However, Native-PAGE produced a dominant band just above the 20 kDa marker, indicative of a dimeric protein (Figure 4B). Weak bands were visible for higher molecular weight species at sizes broadly consistent with tetrameric and octomeric protein. The relative proportions of oligomeric CCD3 was unaffected by storage at −80°C and subsequent thawing suggesting that the minimal coiled-coil motif is stable (Figure 4B). The difference in predicted mass observed with these techniques likely results from the influence of molecular charge in the Native-PAGE. CCD3 is 22.7% acidic, negatively charged, residues, but only 13.4% basic, positively charged, residues. Consequently its migration through the gel matrix will be increased.

AUC and crystallography show that yeast ATG16 is a dimer [16]. As SEC can overestimate the molecular weight of non-globular proteins [21], and because the results of Native-PAGE may be overly affected by molecular charge, we sought alternative confirmation of the oligomeric status of CCD3. We used nanospray Electrospray Ionisation Mass Spectroscopy (ESI-MS), a well established reliable technique for the study of the stoichiometry and interactions of non-covalent complexes in the gas phase [22]–[26]; and also AUC, the gold-standard for determination of protein molecular weight independently of protein shape and charge. Both approaches indicated that the human ATG16L1 was a dimer in solution. ESI-MS identified a single species with a measured molecular weight of 22,687±66 Daltons (Figure 4C); whilst AUC measured the molecular weight to be approximately 23.7 kDa with an rmsd of 0.006 (Figure 4D). Hence, just like the yeast ATG16 coiled-coil, the isolated human ATG16L1 coiled-coil exists as a dimer and is likely to be involved in homomeric interactions during the creation of the multiprotein complexes involved in autophagosome formation. This would then allow the ATG5 binding motif and WD40 motifs to recruit the appropriate interaction partners to facilitate multiprotein complex formation.

### The coiled-coil domain of ATG16L1 is highly conserved across vertebrate evolution

**Figure 5:**
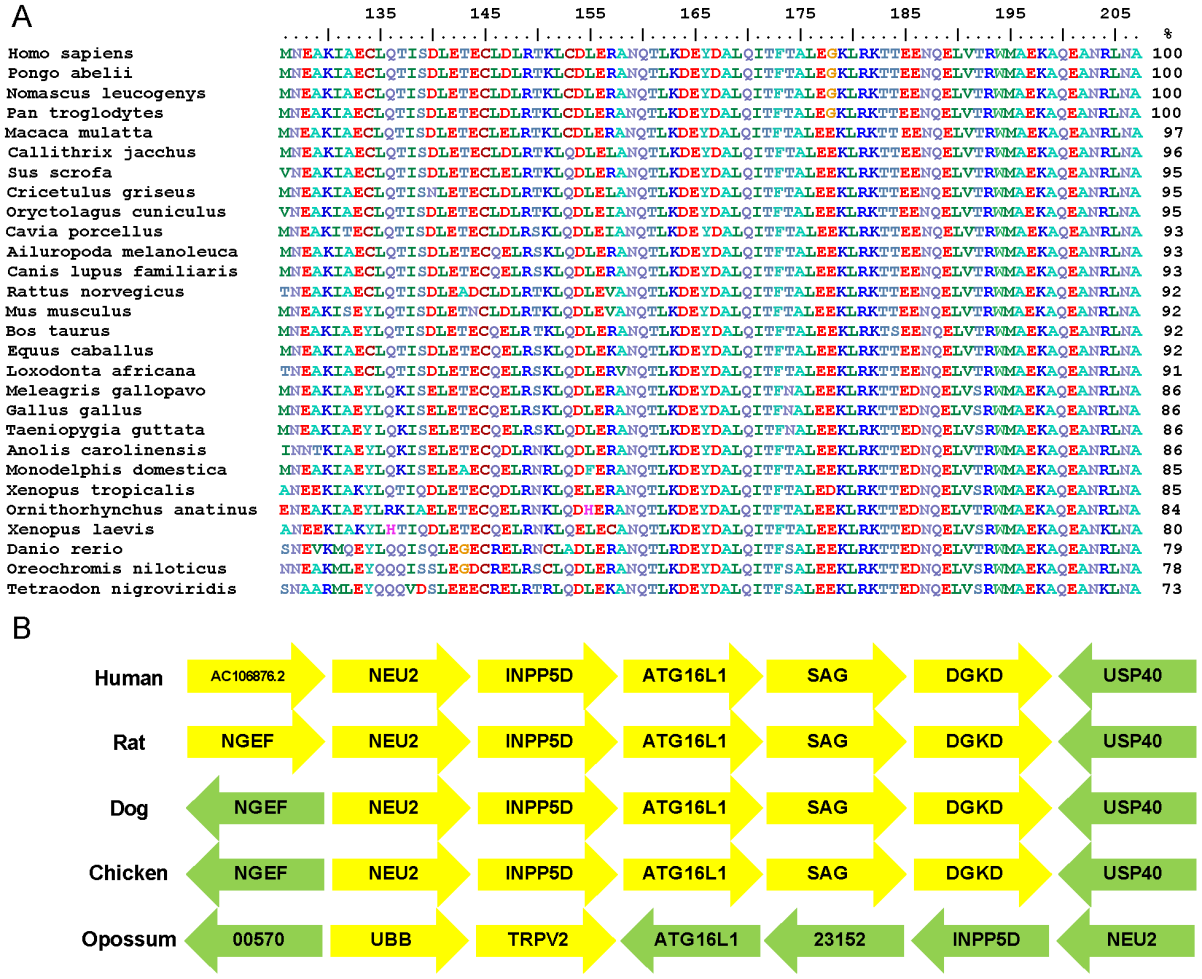
The coiled-coil of ATG16L1 is highly conserved across vertebrates. (A) Cross-species alignment of the coiled-coil (human residues M126 – A207) of ATG16L1 reveals levels of sequence identity with the human sequence of between 73% and 100%. The percentage identity listed is in comparison with the human sequence. The common names and database identifies for each sequence are listed in Materials and Methods. (B) The syntenic position of *Atg16L1* is well conserved across species. The three adjacent upstream and downstream genese to *Atg16L1* are displayed. Genes are denoted by individual blocks; yellow indicates a position on the forward strand and green a position on the reverse strand. Gene identities are as follows: ATG16L1 – autophagy related protein 16 isoform 1; SAG – S-antigen, retina and pineal gland; DGKD – diacylglycerol kinase delta; USP40 – ubiquitin specific peptidase 40; INPP5D – inositol polyphosphate-5-phosphatase; NEU2 – sialidase 2; NGEF - neuronal guanine exchange factor; TRPV2 – transient receptor potential cation channel, subfamily V, member 2; UBB – ubiquitin B; AC106876.2 – uncharacterised; 00570 (ENSMODG00000000570) – uncharacterised; 23152 (ENSMODG00000023152) – uncharacterised, BLASTp indicates a possible ortholog of glyceraldehydes 3-phosphate dehydrogenase.

Cross-species comparisons of protein sequences can provide insight into the functional and structural importance of particular regions of the protein. We used human ATG16L1 to perform a BLASTp search of the non-redundant protein database. From the resulting hits we extracted a broad range of vertebrate orthologs of ATG16L1 including representatives of fish, reptiles, amphibians, birds, marsupials and mammals. Orthologs were aligned using MUSCLE and the alignment manually refined to remove incomplete sequences before trimming to sequences aligned with the coiled-coil region (residues 126 to 207) of human ATG16L1 (Figure 5A). Yeast ATG16 and human ATG16L1 coiled-coil domains show limited conservation [16]. However, the coiled-coil region of vertebrate ATG16L1 aligned with an exceptionally high level of conservation (73–100% identity; Figure 5A). These levels of identity are broadly retained across the full length of the protein (data not shown). The greatest divergence was observed, as expected, between primate and fish proteins. For example, *Homo sapiens* and *Tetraodon nigroviridis* showed 73% identity. The human sequence was completely identical to that of the Sumatran orangutan, the northern white-cheeked gibbon, and the chimpanzee (Figure 5A). The sequences of the dog and the giant panda were also identical to one another. When comparing only sequences from placental mammals the range of identity increased to between 91% and 100%. The high level of sequence identity is indicative of a key functional role for ATG16L1, and the coiled-coil domain in particular. A comparison of the syntenic position of ATG16L1 across a diverse subset of representative species (human, rat, dog, chicken, opossum) also showed a high level of cross-species similarity (Figure 5B). The two genes immediately flanking *Atg16L1* in the upstream and downstream orientation are identical in the human, rat, dog and chicken. The third upstream gene differs in humans which possesses AC106876.2, an uncharacterised gene encoding an 86 amino acid protein; whilst the other three species have the gene for neuronal guanine exchange factor (NGEF). However, NGEF is the next upstream gene in humans, further supporting the similarities between the syntenic position of the species. Even the more distantly related opossum shares two genes, and also has NGEF in the fourth downstream position. Together this data indicates that the *Atg16L1* gene has retained a fairly well conserved genomic position and undergone little amino acid variation across species; characteristics consistent with its crucial role in autophagosome formation.

## Methods

### Plasmids and Gateway® cloning

Potential domain boundaries of the human ATG16L1 coiled-coil region were identified using the sequence alignments of Fujioka and colleagues [16]. Full-length ATG16L1 and fragments corresponding to residues 1–207 (CCD1) and 126–207 (CCD2) were PCR amplified, using Gateway® compatible primers, from pCMV-FLAG-ATG16L1 (a kind gift from Dr Dunecan Massey, Cambridge Institute for Medical Research). Forward primers contained an N-terminal TEV cleavage site; reverse primers a Flag-6xHis tag. Invitrogen Gateway® cloning technology enabled insertion of each PCR product into the destination vectors pDest15 (GST), pDEST HisMBP, pDEST 544 (HisNusA).

### Expression and purification of human ATG16L1 constructs

Human ATG16L1 expression was screened in *E. coli* Rosetta™ 2 cells (Merck Millipore) induced with 1 mM Isopropyl β-D-1-thiogalactopyranoside (IPTG) at 20°C for 14 hours in 2 ml Luria Broth (LB; 10 g NaCl, 10 g Tryptone and 5 g Yeast Extract per Litre deionised water) supplemented with 100 µg/ml of Carbenicillin and 34 µg/ml Chloramphenicol. Cells were pelleted by centrifugation (16,300×g, 5 min); and lysed with 100 µl of BugBuster (Novagen). Soluble and insoluble fractions were separated by centrifugation (16,300×g, 5 min), mixed with SDS-PAGE sample buffer and analysed using 10% SDS-PAGE.

Large-scale protein expression was performed in 1 litre LB as above. Following centrifugation (4,000×g, 20 min) pellets were resuspended in lysis buffer [1× phosphate buffered saline (PBS) (0.137 M, NaCl, 2.7 mM KCl, 4.0 mM Na_2_HPO_4_), 10 mM Dithiothreitol (DTT), 30 mg/ml Lysozyme and 1× Protein Inhibitor Cocktail V (Calbiochem)], sonicated on ice and centrifuged (48,384×g, 30 minutes). Soluble fusion protein was recovered using glutathione sepharose beads (GE Healthcare) at 4°C for 2 hours with rolling. Beads were placed in a gravity flow column and washed 5 times with (then resupsended in) 20 ml 1× PBS, 1 mM DTT, 1× Protein Inhibitor Cocktail V. One tenth volume of Tobacco Etch Virus (TEV) protease was added and incubated for fourteen hours at 4°C. Cleaved protein was eluted in 2 ml fractions. Eluates were pooled and purified further using either anion exchange or hydrophobic interaction chromatography (HIC). For anion exchange pooled eluates were diluted 10-fold in IE diluent buffer (25 mM Tris pH 7.0, 1 mM DTT), then applied to a Resource™ Q anion exchange column (GE Healthcare) pre-equilibrated in IE diluent buffer. Protein was eluted over 30 column volumes with a 0–50% gradient of 25 mM Tris pH 7.0, 1 M NaCl and 1 mM DTT. For HIC elutions from the glutathione resin were diluted 20-fold in HIC binding buffer (2 M Ammonium sulphate, 25 mM Tris pH 8.0) and applied to a 5 ml HiTrap™ Butyl FF column (GE Healthcare) pre-equilibrated with HIC binding buffer. Loosely bound protein was removed with a 0–40% gradient of elution buffer (25 mM Tris pH 8.0) before purified recombinant protein was recovered over 18 column volumes with a 40–100% gradient of elution buffer. Purified CCD3 was buffer exchanged into 25 mM Tris pH 7.0, 100 mM NaCl and 1 mM DTT through a HiPrep™ 26/10 Desalting column (GE Healthcare) before concentration and further analysis.

### TEV Protease Expression and Purification

*E. coli* Rosetta™ 2 cells were transformed with TEV expression plasmid (a kind gift from Prof N Gay, University of Cambridge). Expression of recombinant protein was induced with 1 mM IPTG for 14 hours at 20°C. Cells were pelleted (4,000×g, 30 min); resuspended in 50 ml of TEV lysis buffer (300 mM NaCl, 25 mM Sodium phosphate, 20 mM Imidazole, 0.1% Triton (v/v) and 5 mM β-mercaptoethanol); lysed by sonication; centrifuged (48,384×g, 30 min); and the soluble extract incubated with His-Select® Nickel Affinity Gel (Sigma Aldrich) for 3 hours. Nickel Affinity Gel was applied to a gravity flow column, washed five times with 20 ml of TEV lysis buffer, and recombinant TEV eluted using TEV lysis buffer supplemented with 200 mM Imidazole and 5% glycerol (v/v). TEV was further purified using a Hiload™ 16/60 Superdex™ 75 column (GE Healthcare) equilibrated with 300 mM NaCl, 25 mM Sodium phosphate and 5% glycerol (v/v). Purified protein was collected in 2 ml elutions.

### Analytical Gel Filtration

6 mg of purified protein was loaded onto a Superdex™ 200 10/300 GL column (GE Healthcare) equilibrated with 25 mM Tris pH 7.0, 150 mM NaCl and 1 mM DTT. The following molecular standards were used for estimation of molecular weight: Aprotinin (6.5 kDa), Cytochrome C (12.4 kDa), Carbonic Anhydrase (29 kDa), Albumin (66 kDa), Alcohol dehydrase (150 kDa), β-amylase (200 kDa) and Blue Dextran (2,000 kDa; all from Sigma Aldrich).

### Native-PAGE

The native conformation of CCD3 was analysed using Novex® 4–20% Tris-glycine native gels (Life Tehnologies). Samples were mixed with Novex® native Tris-glycine 2× sample buffer (Life Technologies) and 1 µg of protein was loaded per lane. Gels were run at 125 V for 1.5–2 hours using Novex® Tris-glycine native running buffer (1×) (Life Technologies). Nativemark (Life Technologies) protein size standards specific for native gels were run in parallel.

### Mass spectroscopy

CCD3 was buffer exchanged using Biospin 6 micro-spin columns (Biorad) into 200 mM ammonium acetate, pH 7. Analysis of the oligomeric nature of CCD3 was performed using previously described protocols [22]. In summary, sample ionisation was achieved by nano-electrospray ionisation using in-house prepared gold-coated glass capillaries. A Synapt-HDMS mass spectrometer (Waters) fitted with a 32K quadrupole and set in positive ion mode was used to acquire mass spectra. Nitrogen was used in the IMS T-wave cell and argon in the trap/transfer T-wave region. Raising the collision energy in the trap T-wave region led to collision-induced dissociation and ion activation. Calibration was achieved with Cs_n_I_n−1_^+^ clusters. Data processing and analysis used Masslynx.

### Analytical Ultracentrifugation (AUC)

Sedimentation velocity experiments were conducted with an Optima XLI (Beckman Coulter) using an An60 Ti four-hole rotor. Standard double-sector Epon centerpieces equipped with sapphire windows contained 400-µL of CCD3FH at 1.5 and 0.5 mg/ml. Interference data were acquired in the continuous mode without averaging and with radial increments of 0.003 cm. The density and viscosity of the buffer and the partial specific volume of CCD3FH were calculated using Sednterp [27]. Multicomponent sedimentation coefficient distributions were modelled using Sedfit [28].

### Circular Dichroism

Proteins were buffer exchanged into 25 mM sodium phosphate pH 7.0, 100 mM sodium fluoride, 1 mM TCEP; centrifuged (16,000× g, 10 minutes, 4°C); and 400 µl loaded into a 1.0 mm quartz cuvette at an OD_280_ reading of ∼0.4. CD analysis was performed using an Aviv model 4.0 CD spectrophotometer. Dichroweb [29] was used to convert machine units to mean residue ellipticity [θ] prior to graphical presentation with GraphPad Prism 5. SELCON3, via Dichroweb, was used to determine the proportional secondary structure composition.

### Bioinformatics, database sequences and secondary structure prediction

Protein sequences for database searching and bioinformatics were extracted from the NCBI depositions for human ATG16L1 isoform 1 (NP_110430.5) and *Saccharomyces cerevisiae* ATG16 (NP_013882.1). Protein data bank accession codes used in figure preparation and homology modelling were as follows: 3A7O – *S. cerevisiae* coiled-coil domain [16]; 4GDK – human ATG16L1 ATG5 binding domain [4]; 2DYO – *S. cerevisiae* ATG16 ATG5 binding domain [3]. The secondary structure of human ATG16L1 residues 126–207, corresponding to the CCD3 construct, was predicted using PSIPRED [30]. Protein alignments were performed using MUSCLE and manually refined; CLustal W2 was used to calculate pairwise sequence identity. The sequences used in the cross-species alignment were: Homo sapiens (Human, NP_110430.5), Pongo abelii (Sumatran orangutan, NP_001125757.1), Nomascus leucogenys (Northern White-cheeked gibbon, XP_003278606.1), Pan troglodytes (Chimpanzee, XP_001150112.1), Macaca mulatta (Rhesus macaque, AFE78970.1), Callithrix jacchus (Common Marmoset, XP_002749988.2), Sus scrofa (Pig, NP_001177201.1), Cricetulus griseus (Chinese Hamster, EGW02492.1), Oryctolagus cuniculus, (European rabbit, XP_002721481.1), Cavia porcellus (Guinea Pig, XP_003474455.1), Ailuropoda melanoleuca (Giant Panda, XP_002918010.1), Canis lupus familiaris (Dog, XP_850664.1), Rattus norvegicus (Brown Rat, NP_001102279.2), Mus musculus (Mouse, BAC55091.1), Bos taurus (Cow, DAA30946.1), Equus caballus (Horse, XP_001915817.1), Loxodonta africana (African Bush Elephant, XP_003418005.1), Meleagris gallopavo (Wild Turkey, XP_003209019.1), Gallus gallus (Chicken, XP_422568.2), Taeniopygia guttata (Zebrafinch, XP_002197297.1), Anolis carolinensis (Carolina Anole Lizard, XP_003218430.1), Monodelphis domestica (Grey short-tailed opossum, XP_001373496.2), Xenopus tropicalis (Western clawed frog, CAJ82169.1), Ornithorhynchus anatinus (Platypus, XP_001520939.2), Xenopus laevis (African clawed frog, NP_001091437.1), Danio rerio (Zebrafish, NP_001017854.1), Oreochromis niloticus (Nile Tilapia, XP_003457870.1), Tetraodon nigroviridis (Green-spotted pufferfish, CAG02123.1).

## Conclusion

There is strong functional and evolutionary evidence for the importance of autophagy. Here we have shown that the coiled-coil region of vertebrate ATG16L1 is highly conserved and just like its yeast ortholog likely to exist as a homodimer to facilitate ATG16L1 self-self interactions. ATG16L1 is a crucial component of the inter-protein scaffold required for formation of the autophagosome. This work lays the foundation for future structural studies to enhance our understanding of how ATG16L1 interacts with itself and other proteins, which in turn could help the modulation of autophagy for therapeutic purposes.
